# Effect of Gratitude on Benign and Malicious Envy: The Mediating Role of Social Support

**DOI:** 10.3389/fpsyt.2018.00139

**Published:** 2018-05-07

**Authors:** Yanhui Xiang, Xiaomei Chao, Yanyan Ye

**Affiliations:** ^1^Cognition and Human Behavior Key Laboratory of Hunan and Department of Psychology, Hunan Normal University, Changsha, China; ^2^Department of Psychology, The Chinese University of Hong Kong, Hong Kong, Hong Kong

**Keywords:** gratitude, malicious envy, benign envy, social support, structural modeling

## Abstract

Gratitude has been investigated in various areas in psychology. The present research showed that gratitude had some positive effects on some aspects of our life, such as subjective well-being, life satisfaction, and social relationships. It can also help us relieve negative emotions. However, the existing literature has not studied the influence of gratitude on envy. The present study used structural equation modeling to test the mediating role of social support between gratitude and two types of envy (malicious and benign). We recruited 426 Chinese undergraduates to complete the Gratitude Questionnaire, Malicious and Benign Envy Scales, and the Multi-Dimensional Scale of Perceived Social Support. Results showed that gratitude positively predicted benign envy and negatively predicted malicious envy. In addition, the indirect effect of gratitude on two types of envy via social support was significant. These results revealed the direct relationship between gratitude and malicious/benign envy, and the mediating effect of social support, which will contribute to find effective measures to inhibit malicious envy and promote benign envy from the perspective of cultivating gratitude and increasing individuals' social support.

## Introduction

Gratitude, a kind of positive emotion, has drawn much attention from various psychological perspectives, including personality, social emotion, and clinical psychology (1, 2). Gratitude can be defined as a complex subjective feeling including wonder, thankfulness, and even appreciation in one's life (3, 4), which is a kind of positive energy and has a broad effect in our lives. For example, studies have suggested that gratitude could affect individual subjective well-being and life satisfaction (5–7), strengthen social relationships (8), and elicit prosocial behavior (9–11). At the same time, gratitude could also inhibit negative emotions. For example, researchers have demonstrated that higher levels of gratitude could lead to lower levels of stress or depression (12). Another study also suggested that higher levels of gratitude corresponded with lower levels of envy (13). These studies suggest that gratitude may not only related to positive emotions, but also related to negative emotions such as anxiety, depression, and envy. However, it is important to know how to reduce negative feelings as well as how to enhance positive feeling. The present study focuses on exploring how gratitude affects envy. Until now, there has been no further research exploring how gratitude affects envy, let alone how gratitude affects two quite different types of envy, benign and malicious envy. In the present study, we explore the relationship between gratitude and two types of envy (benign and malicious) and how social support mediates such relationships.

Indeed, some studies have explored the relationship between gratitude and dispositional envy. For example, gratitude was found to have significant negative correlation with dispositional envy (2, 13, 14). The reason may be attributed to the different emotional components between two emotions. Dispositional envy, as a kind of complex negative emotion characterized by resentment, inferiority, longing, and frustration, arises when the self realizes it lacks another's superior quality, achievement, or possessions (15–17). In contrast to envy, gratitude is a complex positive emotion including wonder, thankfulness, appreciation, and so on (3, 4), which is probably incompatible with envy (13, 14). McCullough et al. (13) further demonstrated that grateful people did not focus on acquiring and maintaining possessions and wealth; instead, and they focused on savoring positive experiences and outcomes. Contrary to gratitude, envious people tend to focus on acquiring others' possessions. There is no doubt that people with gratitude would focus on positive contributions of others to their well-being in social comparison rather than to get others' possessions. Therefore, gratitude may have negative correlation with envy.

The rationale under the relationship between gratitude and envy is clear from the above literature. However, in recent years, envy have been classified as explicitly malicious and benign (16–18). Therefore, it is necessary to further explore the relationship between gratitude and the two types of envy. The classification of two types of envy is based on the cognition and motivation function of envy. For example, benign envy elicits individual self-elevating motivation, while malicious envy leads to the tendency of slander or revenge against others (19). Crusius and Lange (20) further demonstrated that benign envy had significant positive correlation with motivation of hope for success, and malicious envy had significant positive correlation with motivation of fear of failure. Although McCullough et al. (2, 13) demonstrated the significant negative correlation between gratitude and dispositional envy, the questionnaire they used on dispositional envy was adopted from Smith et al.'s study (21). Some researchers pointed out that the Dispositional Envy Scale (DES) may represent largely malicious envy, rather than benign envy (17). Therefore, we suggest that gratitude can significantly and negatively predict malicious envy, but this is not true for benign envy. According to condition-elicited benign envy, benign envy is often elicited if one person's advantage is evaluated as subjectively deserved and if the envier perceives high control over personal outcomes (19). If one person tends to have higher levels of benign envy, that person also tends to focus on the positive aspect of the advantages the envied person had over him or her in the context of social comparison. This characteristic is consistent with dispositional gratitude, as described by McCullough et al. (13). Therefore, we hypothesize that gratitude will significantly and positively predict benign envy.

In addition, previous studies indicated that gratitude was closely related to prosocial behavior (9–11, 22). For example, Fredrickson (23) pointed out that gratitude could reflect, motivate, and reinforce social actions in both givers and gift recipients. Therefore, people with higher levels of gratitude may also obtain more social resources, especially social support from others. Some empirical studies have further supported this hypothesis. For example, people with higher levels of gratitude are found to be more likely to perceive and receive greater social support from others, including family, friends, or even strangers (12, 24). Therefore, we hypothesize that gratitude may also positively and significantly predict social support. Regarding social support, it has been identified one of the most promising mediator between gratitude and life satisfaction, which is closely related to mental health (7). Previous studies have also directly demonstrated that social support has a close connection to mental health (25). Specifically, individuals with higher levels of social support may have better mental health. On the contrary, those with lower levels of social support may show more negative emotions, such as depression, anxiety, and hostility, and even express more aggressive behavior (26, 27). However, the presence or absence of hostility is the key characteristic to distinguish between the two types of envy. Smith and Kim (27) suggested that malicious envy is a hostile emotion and has hostile nature. As we mentioned before, envious people typically report depressive, unhappy feelings (21), also hostility. These negative emotional states appear to affect mental health (28–30). It is possible that people with higher level envy doesn't receive and feel the benefits of social support because of their hostile attitudes and behaviors. (31–33). Actually, evidence showed that envy with hostility (malicious envy) was significantly negatively related to social support (32, 33). The reason is that lower social support may mean more hostility or more sensitivity during social interaction or comparison, it is easy for these individuals to experience malicious envy. Similarly, we speculate that higher levels of social support may lead to less hostility in social comparison, which may result in higher levels of benign envy. According to the above analyses, we hypothesize that social support may play a mediating role between gratitude and the two types of envy.

Thus, the present study focuses on exploring the relationship between gratitude and benign/malicious envy as well as the mediating role of social support. Based on the review literature above, we propose the following hypotheses: (1) Gratitude can significantly and positively predict benign envy and negatively predict malicious envy. (2) Social support can positively predict benign envy and negatively predict malicious envy. (3) Social support plays a positive mediating role in the relationship between gratitude and benign envy and a negative mediating role in the relationship between gratitude and malicious envy.

## Materials and methods

### Participants and procedure

Four hundred twenty-six Chinese undergraduates from South China Normal University and Jinan University were recruited randomly. The basic demographic characteristics were shown as follows: 142 men, 284 women; mean age = 20.63 ± 1.85; age range = 18–26 years. The present study was approved by the Academic Committee of the School of Psychology at South China Normal University. All participants were provided with written informed consent before the study and were allowed to leave whenever they felt uncomfortable. It took around 40 min for every participant to finish the following tests.

### Measures

#### Gratitude

This variable was measured by the Gratitude Questionnaire (GQ-6, (13)). This scale includes 6 items with each item rated on a 7-point Likert scale. Higher scores indicate higher levels of gratitude. Many studies have indicated that the Chinese version of the GQ-6 is a reliable and valid measurement (7, 34). Cronbach's alpha coefficient was 0.81 in the present study.

#### Malicious and benign envy

The two scales were developed by Lange and Crusius (17). Each scale consists of 5 items, each scored on a 6-point Likert scale. In this study, the malicious and benign envy scales showed adequate internal reliability (the former Cronbach's alpha was 0.85, the latter was 0.73).

#### Multi-dimensional scale of perceived social support (MPSS)

This scale was developed by Zimet et al. (35). There are 12 items scored on a 7-point Likert scale. This scale consists of three subscales: family's support, friends' support, and others' support. This scale has been used widely among Chinese people (36–38). In the present study, Cronbach's alpha coefficient for the MPSS was 0.90.

### Data analysis

To test whether the indicators represent the latent variables, a measurement model was tested at the beginning in AMOS 17.0. In addition, we also divided the items for gratitude and benign and malicious envy in the MPSS into two or three parcels to serve as indicators of the factors using an item-to-construct balance approach (39). To judge the model's goodness of fit, we chose the Chi square statistic, standardized root-mean-square residual (SRMR), root-mean-square error of approximation (RMSEA), and comparative fit index (CFI) as the indicators (40). In addition, we used the Akaike Information Criterion (AIC) to judge which model demonstrated better fit (41). The expected cross-validation index (ECVI) was used to evaluate potential for replication (42).

## Results

### Measurement model

Latent variables in the measurement model included gratitude, benign envy, malicious envy, and MPSS. Results showed that the data fit well into the measurement model [χ^2^_(21, N = 426)_ = 15.245, *P* = 0.002; RMSEA = 0.052; SRMR = 0.0373; CFI = 0.984]. In addition, factor loadings of all the latent variables were significantly correlated (*P* < 0.001), which suggested that latent variables well represented the observed variables. Moreover, all latent variables were significantly related. Means, standard deviations, and correlations between gratitude, benign envy, malicious envy, and social support are shown in Table 1.

**Table 1:** Descriptive statistics and zero-order correlations for all measures.

**Measure**	**M**	**SD**	**1**	**2**	**3**	**4**
1 GQ	31.89	5.77	1			
2 BE	23.44	3.68	0.25^**^	1		
3 ME	11.84	4.73	−0.33^**^	−0.29^**^	1	
4 MPSS	62.49	11.17	0.55^**^	0.27^**^	−0.27^**^	1

*GQ, Gratitude Questionnaire; BE, Benign envy; ME, Malicious Envy; MPSS, Multi-Dimensional Scale of Perceived Social Support*.

** *p < 0.001*.

### Structural model

**Figure 1:**
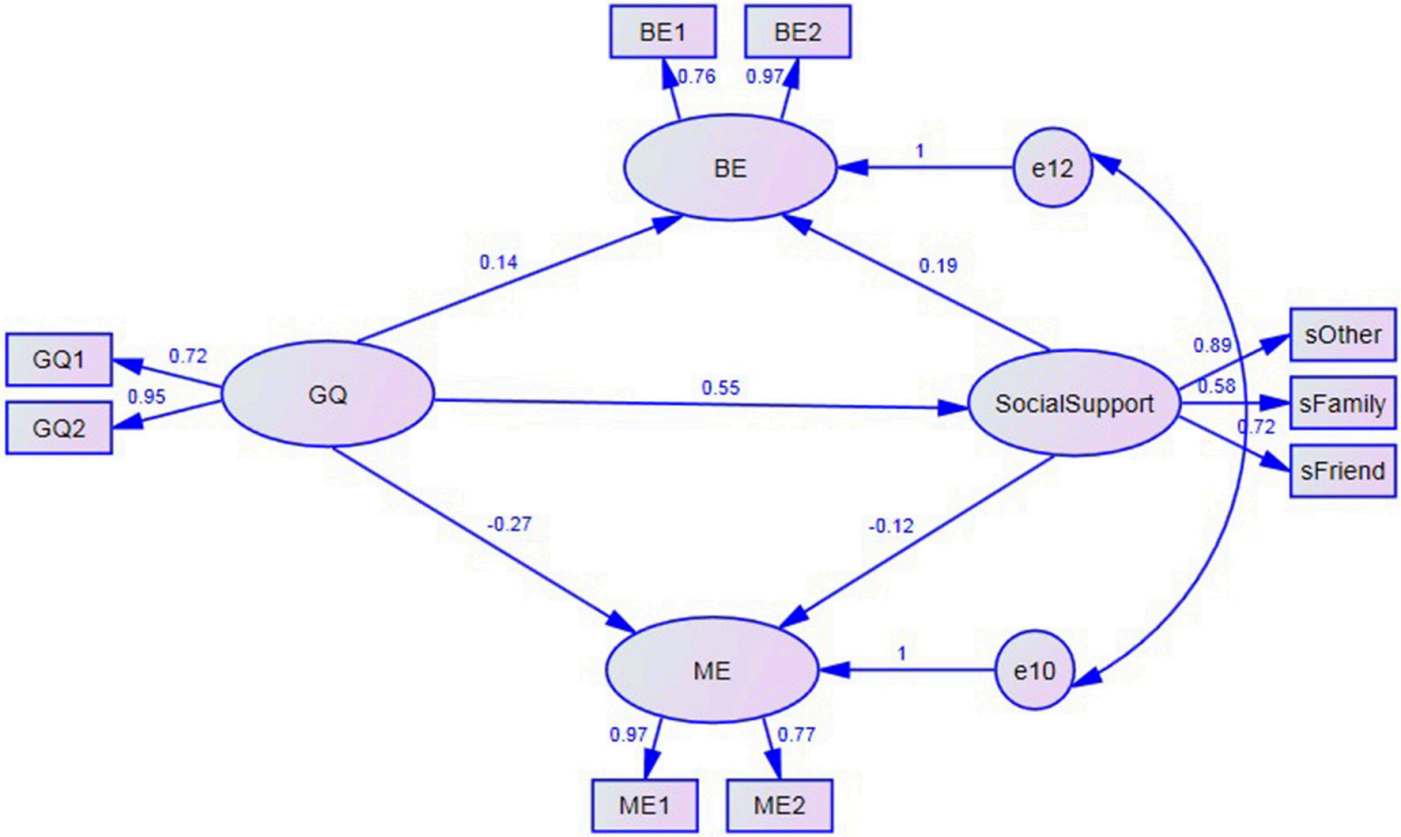
The mediation model. Factor loadings are standardized. BE1 and BE2 are two parcels of benign envy; ME1 and ME2 are two parcels of malicious envy; GQ1 and GQ2 are two parcels gratitude; sOther, sFamily and sFriend are subscales of the Multi-Dimensional Scale of Perceived Social Support.

When lacking the mediator (social support), gratitude (predictor) could significantly predict benign (β = 0.20, *P* < 0.01) and malicious envy (β = −0.28, *P* < 0.001) (criterion), respectively. We then set Model 1, in which gratitude directly predicted benign and malicious envy while also indirectly predicting those two variables through social support. Results showed that all indices except RMSEA were good indices (Table 2) [$\chi_{\left(22,\;\text{N}=426\right)}^{2}$ = 61.903, *P* < 0.001; RMSEA = 0.065; SRMR = 0.0572; and CFI = 0.974]. Further, we found that benign envy and the error terms of malicious envy were admissibly correlated. Thus, we constructed Model 2 based on Model 1. Results showed that this model had significantly good fit for the observed variables [$\chi_{\left(21,\;\text{N}=426\right)}^{2}$ = 45.245, *P* < 0.001; RMSEA = 0.052; SRMR = 0.0373; and CFI = 0.984]. Compared with Model 1, we found that Model 2 had a much smaller [Δχ^2^_(1, N = 426)_ = 16.657, *P* < 0.001] and a smaller AIC, suggesting that Model 2 has a better fit than Model 1. Thus, we used Model 2 as the final testing model (See Figure 1).

**Table 2:** Fit indices between Model 1 and Model 2.

	**χ^2^**	**df**	**RMSEA**	**SRMR**	**CFI**	**AIC**	**ECVI**
Model 1	61.903	22	0.065	0.0572	0.974	107.903	0.254
Model 2	45.245	21	0.052	0.0373	0.984	93.245	0.219

*RMSEA, root-mean-square error of approximation; SRMR, standardized root-mean-square residual; CFI, comparative fit index; AIC, Akaike information criterion; ECVI, expected cross-validation index*.

Further, we used a bootstrap estimation procedure to investigate the robustness of this mediation effect. Results indicated that a 95% confidence interval was significantly correlated to the mediation effect. As we can see in Table 3, gratitude significantly and indirectly influenced benign (95% confidence intervals [0.03~0.11]) and malicious envy (95% confidence intervals [−0.10~−0.02]) through the mediating variable social support.

**Table 3:** Standardized indirect effects and 95% confidence intervals.

**Pathways**	**Estimate**	**Lower**	**Upper**
Gratitude-Social Support-Benign Envy	0.03	0.03	0.11
Gratitude-Social Support-Malicious Envy	0.03	−0.10	−0.02

### Gender difference

We used four latent variables to test for difference between genders. Results showed that there was no significant gender difference in malicious envy [*t*_(424)_ = −0.076, *P* = 0.939] and gratitude [*t*_(424)_ = −1.391, *P* = 0.165]. However, we found significant gender differences in social support [*t*_(424)_ = −2.437, *P* = 0.015] and benign envy [*t*_(424)_ = −2.117, *P* = 0.035], in both of which female participants scored higher than male participants. Based on this, we further explored the robustness of gender differences in the structural equation model that we found.

We used a multi-group analysis to investigate the path coefficients in the model of gender differences. Based on keeping the basic parameters (factor loadings, error variances, and structure covariances) stable, we constructed two models. One allowed for free estimations of paths across genders in the two models while the other limited the paths equaled in two models. Results showed significant differences in [${\Delta \chi }_{\left(14,\;\text{N}=426\right)}^{2}$ = 35.303, *P* < 0.001]; however, the two models reached the standard of fitness when we compared other parameters in the two models (see Table 4). Thus, in general, deformable models of parameter limitations in multiple groups are acceptable. In addition, we used critical ratio of differences (CRD) as an indicator in investigating the difference between the standard errors of two sexes. According to the decision rule, CRD > 1.96 means the two parameters are significantly different at a significant level of *P* < 0.05. The results showed that the structural path from GQ to social support had significant difference (CRD = 2.467, *P* < 0.05). Specifically, the path coefficient in male participants was β = 0.635 (*P* < 0.001) while the path coefficient in female participants was β = 0.445 (*P* < 0.001). This result indicates the positive effect of gratitude in social support is significantly greater in male than in female participants.

**Table 4:** Constrained and unconstrained structural paths across genders.

	**χ^2^**	**df**	**RMSEA**	**SRMR**	**CFI**	**AIC**	**ECVI**
Unconstrained SP	91.569	47	0.047	0.0788	0.972	177.569	0.419
Constrained SP	126.311	61	0.050	0.0801	0.959	184.311	0.435

*SP, structural path; RMSEA, root-mean-square error of approximation; SRMR, standardized root-mean-square residual; CFI, comparative fit index; AIC, Akaike information criterion; ECVI, expected cross-validation index*.

## Discussion

The present study aimed to test the relationship between gratitude and benign/malicious envy and the mediating role of social support. The result revealed that gratitude could predict benign envy positively and malicious envy negatively. Furthermore, the indirect effect of gratitude on the two types of envy via social support was significant. These results first directly revealed the relationship between gratitude and two different kinds of envy and the mediating effect of social support, which contributed to effective measures to inhibit malicious envy and promote benign envy from the perspective of cultivating gratitude and increasing individuals' social support.

According to the result of a correlation analysis, gratitude had significant and negative correlation with malicious envy, which was also confirmed by the regression coefficient in the structure model. This result is consistent with the results of previous studies (13, 14), which also found this negative relationship between gratitude and envy with hostile emotion. On the other hand, consistent with our hypothesis, we also found that gratitude was related significantly and positively to benign envy, which was in accordance with the regression coefficient in the structure model. Two opposite results indicated that the relationship between gratitude and envy, which has been demonstrated by previous studies, should be distinguished according to different types of envy. In particular, higher levels of gratitude indicated lower levels of malicious envy and higher levels of benign envy. This can be explained by McCullough et al. (2, 13)—that is, gratitude leads individuals to focus on positive contribution of the envied person in social comparison; therefore, they tend to show lower malicious envy and higher benign envy.

Moreover, in line with our hypothesis, we found gratitude was positively related to social support, which was also consistent with several empirical. Previous studies indicated that gratitude was significantly and positively correlated with social support (7, 12, 43). In addition, we also found that social support was positively related to benign envy and negatively to malicious envy. The former result was consistent with previous studies (32, 33), and the latter supported our previous hypothesis—that is, higher social support may inhibit the hostility of malicious envy, resulting in lower levels of malicious envy. In addition, previous studies also explored the relationship between social support and social pain or perceived control. Some studies found that social support benefited coping with social stress and relieving social pain (44, 45), and many others also demonstrated that higher social support can improve the feeling of perceiving control (46, 47). According to previous studies, control potential was a key factor to distinguish between two kinds of envy; greater control perception tended to be elicited by benign envy, and reduced control perception tended to be elicited by malicious envy (48). Therefore, higher social support may improve the feeling of self controllability in facing stress because of social comparison and then promote benign envy and inhibit malicious envy.

Based on the analyses above, we can also further explain the mediating effect of social support in the final model. There existed two partial mediating relationships as follows: gratitude → social support → benign envy, gratitude → social support → malicious envy. Combined with the direction of the correlation coefficient, we can conclude that grateful people tend to have high levels of social support from others, and, furthermore, higher levels of social support will lead to higher levels of benign envy and lower levels of malicious envy. Social support plays a positive role in the interaction between gratitude and benign envy but plays an inhibitory role in the relationship between gratitude and malicious envy. Whatever promotes benign envy or inhibits malicious envy, the key factor depends on whether higher social support can effectively relieve social pain or strengthen the feeling of self-control. One encouraging thing about this result is, we found social support plays a rather critical role here, which is consistent with previous studies that social support is closely related to mental health (25, 26). And this is a quite practical way to improve both gratitude and benign envy, as well as reducing malicious envy. This provides preliminary evidence for future studies using social support as an intervention, which will definitely contribute to the area of mental health.

In the test of gender difference, we also found that women show more benign envy compared to men. Previous studies explored the gender difference of envy in special fields. For example, Hill and Buss (49) found that female participants showed more envy than male participants when their companions were more attractive than they were, while male participants became more envious than female participants when their peers had higher-quality sex lives. DelPriore et al. (50) further found that the envy-evoking events of the two genders did not stay stable across time. They changed according to major classes of adaptive challenges facing humans over evolutionary time. In our study, whatever the measures of benign envy and malicious envy, which is the general envy and not involved in specific field of social comparison. Therefore, our results may be an extension to the results of the previous study, showing that female participants tended to display more benign envy than male participants but showed no difference in malicious envy. Furthermore, we also found that women have higher levels of social support than men, which is consistent with previous studies (51, 52). However, in the further analysis, we found that male participants with high levels of gratitude tended to get more social support than female participants, which was also found in previous studies (24, 36, 37). Men may get more return of social support from gratitude than women. This may reflect differences of sex roles in society. Although we found these gender differences in particular variables, the final model actually showed no gender difference, demonstrating that the final model was stable across genders.

## Author contributions

YX: Study design, data collection, data analysis, paper writing. YY: Study design, paper writing, paper revising. XC: Data collection, paper revising.

### Conflict of interest statement

The authors declare that the research was conducted in the absence of any commercial or financial relationships that could be construed as a potential conflict of interest.
